# Challenges to the provision of specialized care in remote rural municipalities in Brazil

**DOI:** 10.1186/s12913-022-08805-6

**Published:** 2022-11-22

**Authors:** Fabiely Gomes da Silva Nunes, Adriano Maia dos Santos, Ângela Oliveira Carneiro, Márcia Cristina Rodrigues Fausto, Lucas Manoel da Silva Cabral, Patty Fidelis de Almeida

**Affiliations:** 1 Multidisciplinary Health Institute, Federal University of Bahia, Vitória da Conquista, Bahia Brazil; 2 Federal University of Vale do São Francisco, Petrolina, Pernambuco Brazil; 3 Sérgio Arouca National School of Public Health, Oswaldo Cruz Foundation, Rio de Janeiro, Brazil; 4 Hésio Cordeiro Institute of Social Medicine, Rio de Janeiro State University, Rio de Janeiro, Brazil; 5 Collective Health Institute, Fluminense Federal University, Niterói, Rio de Janeiro, Brazil

**Keywords:** Secondary health care, Access to health services, Rural health services, Comprehensive health care, Brazil

## Abstract

This case study analyses the challenges to providing specialized care in Brazilian remote rural municipalities (RRM). Interviews were conducted with managers from two Brazilian states (Piauí and Bahia). We identified that the distance between municipalities is a limiting factor for access and that significant care gaps contribute to different organizational arrangements for providing and accessing specialized care. Physicians in all the RRMs offer specialized care by direct disbursement to users or sale of procedures to managers periodically, compromising municipal and household budgets. Health regions do not meet the demand for specialized care and exacerbate the need for extensive travel. RRM managers face additional challenges for the provision of specialized care regarding the financing, implementation of cooperative arrangements, and the provision of care articulated in networks to achieve comprehensive care, seeking solutions to the locoregional specificities.

## Introduction

The Brazilian public model of actions and services is based on the Unified Health System (SUS), which is structured by sharing responsibilities among the three spheres of the federation – the Federal Government, states, and municipalities [1].

The SUS is a universal state policy that expanded the Brazilian social protection system from the perspective of conforming to a Welfare State [2]. To this end, it assumes health as a right of all and attribution of the State [3], which, in turn, should provide comprehensive and articulated services at different levels of care – primary health care, specialized health care, and hospital care [4].

Different studies attest to the health success of the SUS and its impact on the quality of life of Brazilians [5–7]. However, the SUS is not only composed of public services but also of a vast network of contracted private services, mainly hospitals and diagnosis and therapy units [1, 8], which are remunerated with health-oriented tax resources [9].

The participation of private service providers in the SUS should be complementary [8] and linked to the public entity’s inability to assure care coverage – universally and comprehensively – to the population of a given health region.

The health regions [10] – select health territories for the integration of health services [11] – are inspired by the classic model of the Dawson Report [12]. Furthermore, they must be planned in the logic of health care networks and organized based on criteria of scale, quality, and access opportunity [13].

In turn, the participation of private institutions in the SUS must occur through agreements and covenants. Funding from several revenues collected by the Federal Government, states, and municipalities must facilitate people’s access to SUS actions and services. It should also not be conditional on people’s ability to pay in advance [14].

Among the main obstacles to ensuring comprehensive care in the SUS are the restricted offer of specialized care – outpatient medical services, diagnostic and therapeutic support, the difficult access in circumstances that allow appropriate use timely, and the recurrent public dependence on agreements with the private sector [9, 15].

Access to specialized care becomes even more critical in small municipalities [16] with rural and remote characteristics [17], in the face of the great distances to urban clusters, insufficient and inadequate number of professionals, and the unavailability of or high costs associated with health transportation [18, 19]. These are common challenges in countries with vast geographical distances and territories with low population density [20].

Specialized care should preferably be offered to guarantee an adequate scale in order to ensure cost-effectiveness and quality of care [21]. In contrast, in the rural population’s daily life, the location of services based exclusively on the economic logic often implies a lack of care [18], loss of timely care [19], and inequalities [22, 23] in large and sparsely populated territories, marked by weak infrastructure and historical absence of social policies.

In Brazil, the guideline of regionalization through health regions has been the organizational strategy to articulate municipalities of the same health territory to share specialized care, concentrating services (by scale and scope) in the region’s host city, with a prospect of combining continuity, integration, and coordination of care [24], with primary health care (PHC) as the preferred gateway [25]. The supply of services and care flows are defined through agreements and pacts between managers of the municipalities underpinning the regional health territory in SUS governance authorities [26].

Brazil has been implementing comprehensive programs [1–3] to address persistent problems in the health sector in remote and rural territories over the last few decades. The Family Health Strategy – a priority PHC model in the country – for example, has positively impacted municipalities in the country’s most impoverished regions [4, 6], making it a highly cost-effective tool to improve health in poor areas directly and indirectly [27].

In the same perspective, recent initiatives to attract and retain general practitioners in underserved areas have also affected the expanded access and impacted care in PHC [28]. However, studies point to paradoxical effects in the use of medical care as a result of this government policy [29, 30].

In the Brazilian semi-arid region, socioeconomically vulnerable populations live in remote rural municipalities (RRM), exposed to non-assistance and loss of therapeutic opportunity due to geographic barriers regarding traveling from the place of residence to the health service or financial constraints [18].

These characteristics should be considered by health system managers when organizing the supply of specialized care – quantity, quality, and location – so that they overcome health inequalities and enable care comprehensiveness [19, 31, 32].

This study analyses the challenges to the provision of specialized care for populations living in RRMs in three health regions of the North-eastern semi-arid region.

## Methodology

This article is nested in a national study on the organization and use of PHC services in Brazilian RRMs [33]. Multiple case studies were conducted in RRMs with a qualitative approach through semi-structured interviews.

In Brazil, the definitions of rural and urban areas gained a typology in 2017 [34], proposed by the Brazilian Institute of Geography and Statistics (IBGE), aligned with Organization for Economic Co-operation and Development (OECD) and European Union methodologies, based on parameters of demographic density, location concerning urban centers, and population size.

In the primary research [33], of which this article is a part, the 323 remote rural municipalities were grouped into six clusters. These clusters were built from the analysis of territorial use, identifying the different ways in which the Brazilian territory was socially and economically distributed.

Santos and Silveira [35] study proposed a Brazilian regional division into “4 Brazils”: the Concentrated Region (South and Southeast); the Region of Recent Peripheral Occupation; the Northeast; and the Amazon, which served as a basis for this analysis.

Initially, the RRMs were plotted on the Brazilian map according to these “4 Brazils”, identifying the areas with the highest concentration of these municipalities. We employed the following variables because of their relevance in the analysis of rural and remote settings worldwide, expressing the declining population, remoteness, and economic capacity: Economic activity profile; GDP composition by different economic activities; Government fund transfer dependence; per capita GDP; population density; proportion of population participating in cash transfer programs.

The characterization of municipalities led to the design of six clusters named: *Matopiba; Norte de Minas; Vetor Centro-Oeste; Semiárido; Norte Águas;* and *Norte Estradas*. These six clusters show distinct spatial logics, agglutinating 97.2% (307) of the 323 RRMs. The sample of municipalities was selected intentionally from defined clusters.

This article analyses the results from four RRMs located in three different health regions circumscribed to the Brazilian semi-arid region (Cluster Semiárido) in Piauí and Bahia. The RRMs of the semi-arid region were characterized through a set of socioeconomic, demographic, and health indicators to define the intentional sample of the study.

Subsequently, we selected municipalities that would approach the “average municipality” considering these variables, which led us to select the municipalities of Rio Grande do Piauí – Piauí state, Morpará, and Ipupiara – Bahia state, besides Pilão Arcado, with outlier characteristics (with population above the average in the area).

The study population consisted of 13 respondents: eight municipal health managers – Municipal Health Secretaries and Primary Healthcare Coordinators (MM); three regional managers (RM), and two state managers (SM). The interviews were held face-to-face, from May to October 2019, lasting from 1 to 2.5 hours, conducted in their respective workplaces, audio-recorded, and transcribed in full.

We proceeded to the thematic content analysis of the material with its respective categorization, description, and interpretation steps to produce the results. Subsequently, we started to compare the statements in the dialectical confrontation of ideas and positions of the subjects, identifying convergences and divergences for critical interpretation. We did not intend to judge each municipality but understand the territorial processes.

## Results

**Fig. 1:**
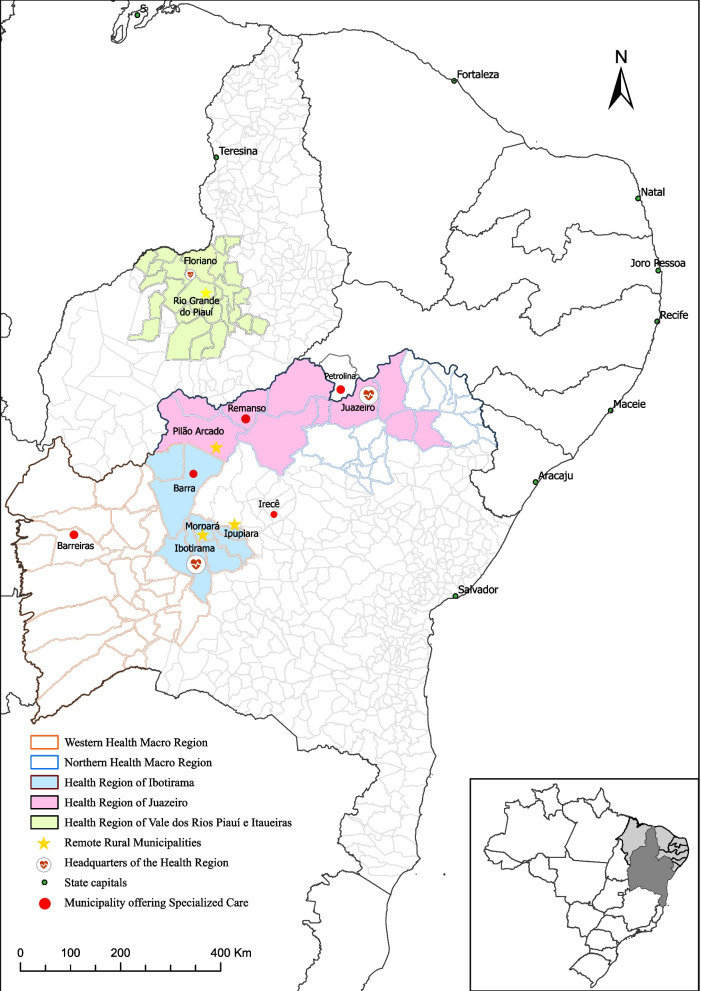
Assistance flows between municipalities and health regions, Semiarid, Brazil, 2019.

The results are organized into two dimensions. Dimension 1 presents the context of health regions: socioeconomic characteristics (Table 1) and the distances between RRMs and main cities that offer specialized care (Fig. 1 and Table 2). Subsequently, Dimension 2 presents the health care points (Table 3) and care regulation in RRMs: from PHC to specialized care. The corresponding empirical data are synthesized with the main findings and their respective speech fragments (Tables 4 and 5).

**Table 1:** Characteristics of Remote Rural Municipalities, Health Regions, Semi-arid, Brazil, 2019

Local	Population [1] 2020	Area (km^2^) [2]	Density (inhab./km ^2^ [2]	Population in rural areas [2] (%)	Extreme poverty [2] (%)	PBF beneficiary population [3] (%)
**Bahia State**	15,324,591	564,732.80	27.14	27.93	12.71	51.93
*Ibotirama Health Region*	*196,095*	*28,667.00*	*7.46*	*33.39*	*31.38*	*78.40*
Ipupiara	10,157	1055.80	9.62	35.60	23.79	76.14
Morpará	8950	2093.90	4.27	33.12	29.89	80.11
*Juazeiro Health Region*	*535,846*	*7467.30*	*10.74*	*26.58*	*24.84*	*72.51*
Pilão Arcado	35,740	11,626.60	3.07	66.44	40.90	74.87
**Piauí State**	3,219,953	251,611.30	12.80	34.23	13.27	58.86
*Vale Rios Piauí and Itaueiras Health Region*	*201,853*	*27,833.10*	*5.39*	*44.55*	*29.42*	*74.81*
Rio Grande do Piauí	6331	636.00	9.95	34.74	31.13	75.78
**Brazil**	211,755,692	8,516,000	23.8	15.67	6.62	21

*Captions:* Population expectation, 2019 | Population benefiting from the *Bolsa Família* Program (PBF, Family Aid Program – National program of cash transfer to people in extreme poverty), 2019 | Population in extreme poverty earn per capita income below R$70.00

*Sources*^1^: *IBGE* Brazilian Institute of Geography and Statistics^2^; *UNDP* Atlas of Human Development in Brazil, Demographic Census^3^;*MDS* Ministry of Social Development, Bolsa Família panel and Unified Registry, 2019

**Table 2:** Distance between Rural Remote Municipalities to the headquarters of health regions/macro-regions and the state capital, Semi-arid, Brazil, 2019

Place of departure		Health region headquarters (distance/time)	Headquarters of the macro-region health region (distance/time)	Capital (distance/time)
Ipupiara (BA)	**Rural area** ^**UR**^	281 km (05 h:25)	489 km (08 h:12)	739 km (12 h:00)
	**Headquarters**	161 km (02 h:25)	369 km (05 h:12)	619 km (09 h:00)
Morpará (BA)	**Rural area** ^**UR**^	108 km (02 h:25)	305 km (05 h:05)	745 km (11 h:20)
	**Headquarters**	86 km (01 h:25)	283 km (04 h:05)	723 km (10 h:20)
Pilão Arcado (BA)	**Rural area** ^**UR**^	299 km (04 h:48)	299 km (04 h:48)	806 km (12 h:40)
	**Headquarters**	281 km (04 h:08)	281 km (04 h:08)	788 km (12 h:00)
Rio Grande do Piauí (PI)	**Rural area** ^**UR**^	163 km (03 h:00)	Does not apply to the state of Piauí*	408 km (06 h:20)
	**Headquarters**	135 km (02 h:00)		380 km (05 h:20)

*Captions: UR* Unpaved road | *PHC* Primary Health Care | * Does not apply to the state of Piauí since the regionalization model consists only of health regions

*Source: DER* Department of Roads and Highways of Bahia (BA) and Piauí (PI). Survey database, based on information from respondents

**Table 3:** Offer of professionals and specialized services selected in Rural Remote Municipalities, headquarters of health regions/macro-regions and states, Semi-arid, Brazil, 2019

Offer of professionals and specialized services	Brazil	Bahia State	*Ibotirama Health Region*	Ipupiara	Morpará	*Juazeiro Health Region*	Pilão Arcado	Piauí State	*Vale Rios Piauí and Itaueiras Health Region*	Rio Grande do Piauí
Population	211,755,692	15,324,591	*196,095*	10,157	8950	*535,846*	35,740	3,219,953	*201,853*	6331
Doctors / thousand inhabitants	**2.3**	**1.7**	*0.5*	*	*	*1.2*	*	**0.9**	*0.4*	*
Nurses / thousand inhabitants	**10.1**	**7.4**	*6.3*	*	*	*11.4*	*	**5.4**	*3.4*	*
General Surgeon	**10,226**	**581**	*4*	*	*	*12*	*	**120**	*2*	*
Pediatrician	**40,905**	**1689**	*2*	*	*	*5*	*	**277**	*	*
Psychiatrist	**8914**	**328**	*1*	*	*	*3*	*	**80**	*	*
Cardiologist	**19,865**	**929**	*2*	*	*	*14*	*	**144**	*1*	*
Cardiovascular Surgeon	**620**	**24**	*	*	*	*	*	**4**	*	*
Pediatric Surgeon	**699**	**24**	*	*	*	*1*	*	**8**	*	*
Plastic Surgeon	**2693**	**80**	*1*	*	*	*3*	*	**13**	*1*	*
Urologist	**2477**	**121**	*3*	*	*	*9*	*	**21**	*	*
Neurosurgeon	**1300**	**44**	*	*	*	*1*	*	**21**	*	*
Emergency Mobile Care Service	**4568**	**460**	*12*	2	1	*18*	1	**141**	*23*	1
Psychosocial Care Center	**3225**	**290**	*5*	*	*	*12*	1	**67**	*5*	1
Specialty Center	**12,227**	**979**	*17*	*	1	*28*	1	***292***	*42*	*
Polyclinic	**2473**	**209**	*	*	*	*6*	1	**20**	*1*	*
Specialized Hospital	**563**	**54**	*	*	*	*5*	–	**13**	*2*	*
Inpatient Beds	**310,675**	**24,288**	*241*	*	*	*861*	30	**6821**	*441*	*
Clinical Beds	**125,880**	**9457**	*71*	6	*	*328*	14	**2720**	*219*	2
Surgical Beds	**73,446**	**6089**	*63*	9	*	*219*	3	**1380**	*72*	*
Obstetric Beds	**38,547**	**3569**	*59*	6	2	*122*	6	**1082**	*73*	*
Pediatric Beds	**36,828**	**3475**	*53*	3	*	*115*	7	**1064**	*49*	*
MRI	**3177**	***184***	*	*	*	*3*	*	***27***	*2*	*
Computed Tomography	**6229**	**286**	*	*	*	*6*	*	**89**	*6*	*
X-Ray	**84,403**	**4806**	*37*	2	4	*111*	1	**513**	*41*	1
Ultrasound	**49,845**	**3552**	*31*	1	2	*46*	1	**493**	*34*	1
Mammography	**5424**	**341**	*4*	*	*	*6*	*	**76**	*5*	*

*Captions:* *Information not available in the National Registry of Health Establishments

*Sources:* SCNES – National Registry of Health Facilities / Ministry of Health

**Table 4:** Summary of results and expressive statements, according to challenges to the provision of specialized care in remote rural municipalities, semi-arid region, Brazil, 2019

Dimension 1	
Summary of challenges	Expressive statements
[Challenge 1] Public manager is the main buyer and payer of specialized services for the population	Some patients go to the regional hospital of Ibotirama, others to the municipal hospital of Barra, and others to Salvador. [...] for example, the municipality of Ibotirama hired a urologist and other doctors from other specialties. Therefore, a private clinic was hired to perform, but the municipality pays a specific agreed value. Concerning MRI and orthopedics, the municipality of Barreiras also contracted a private clinic and we outsourced this service and used it several times. So, we have public to public and public to private, at no cost to the population; no cost, meaning we use funds transferred to us (Municipal health manager 3).
[Challenge 2] Scarce supply and long distances contribute to different arrangements for the provision/acquisition of specialized care	[...] to what extent Juazeiro can meet all this demand. Sometimes, this demand cannot be met [...]. If we say that there is specialized care for all our demands, well, there isn’t. Orthopedics is our great weakness in the mother and child network, and the situation is very complicated for cardiology as well (Regional manager 3). The public service is very outdated and cannot meet what the entire population wants. There has been a lot of private health care, and we understand that it is well served here. The mayor has significantly focused on [public] health; however, private health care has stood out. Perceiving that most of the tests, both quantity and price, are in the private health care in Irecê, then, Ipupiara sends a lot to Irecê. We have even weekly vans making this trip. The fare is expensive; however, it pays off for the population because everyone who goes there likes the private health care service (Municipal health manager 1). Municipalities are very distant from each other. This hinders people’s access to health services, especially regarding medium- and high-complexity problems. It is very challenging for us to structure this flow here in the region. Moreover, within the municipality, some municipalities have locations that stand more than 100 km from the headquarters (Regional manager 1). [...] our territory is in a transitional location, where the care gap in the center-south is enormous. The only reference hospital for medium- and high-complexity is located here in our territory, which is the Floriano hospital. [...] now, the visits [with specialists] are generally in Teresina [...] because some specialties only have two or three professionals for the entire state, so it cannot be sufficient (Regional manager 2).
[Challenge 3] Inequalities to vulnerable populations and the neediest municipalities	For example, we spent nine months with a pregnant woman, and she did only one test, and that was it! Because struggled to access; a pregnant woman, with nausea; she had all the difficulty in the world to go and do these tests [at the headquarters of the health region]. So, we spent nine months with this pregnant woman without a blood count because the mother could not afford to pay [the bus fare] (Municipal health manager 6).

**Table 5:** Summary of results and expressive statements, according to challenges to the provision of specialized care in remote rural municipalities, semi-arid region, Brazil, 2019

Dimension 2	
Summary of challenges	Expressive statements
[Challenge 4] Manager needs to negotiate discounts with private clinics	I think that is a great challenge for the manager. He [health secretary] was a social assistance secretary and knows each person’s profile and tries somehow to filter and prioritize those who are low-income: both economic and clinical triage. If someone can wait, he goes for a normal appointment; however, he tries to help by financing the specialist for low-income people who cannot afford it; that is how it works (Municipal health manager 2).
[Challenge 5] Direct purchase of private services by the public manager strengthens the private network	[...] the municipalities structure specialized care services by hiring professionals. Then, professional go there and provide the service. So, the municipalities do not get paid for it because they do not have a service that SUS can accredit, and everything depends very much on the municipal counterpart (Regional manager 1). [...] It would take six months to have a visit and return with the tests. So, some patients go to SUS for the first visit. When the doctor asks for the tests, they go to the clinic, pay, and then return to show the results to the [SUS] doctor (Regional manager 3). [Specialized care] is much more private in small municipalities. The larger municipalities can have a more adequate structure, as they can resort to some accreditation. However, the management is public and, in the cases of small municipalities, we still have this issue of company contracts, or contracting with private companies or professionals (State manager 1).
[Challenge 6] Faced with large distances, it was often cheaper for the patient to pay out-of-pocket than to travel to the neighboring municipality to receive public care	[...] we have a covenant with the public laboratory of the hospital in Ibotirama. However, people go there, pay the bus fare to collect blood there, the fare is the money for all the tests or the ultrasound. Sometimes, it’s not worth it. Sometimes, people have [public] vacancies, but they prefer not to use them. Then, we can pay it here [in the municipality], through the private laboratory, because it is cheaper even for us (Municipal health manager 2).
[Challenge 7] RRM managers had to offer a supporting point for patients to stay in capitals during treatment	There is a support house in Salvador; several municipalities have an agreement with that house. They have one in Barreiras; some already have one, those municipalities further away have a support house (Regional manager 1). Every municipality has an Out-of-Home Treatment (OHT) car, which they send for treatment outside the municipality. They both come here (health region headquarters) for hemodialysis and other treatments in Salvador (Regional manager 2).
[Challenge 8] The appointment scheduling center was located in the health secretariats at the headquarters of the RRMs	The patient goes through primary care, gets a referral for that specialty, then goes to the secretariat, where he is scheduled in the regulation system; and then, he is referred (Municipal health manager 8)
[Challenge 9] CHWs informed about the appointment scheduling and the delivery of the results of specialized tests	Because some individuals have no way to come [to schedule], the health worker brings the copy of the document. When we make the appointment, we contact them, and usually, when it is a place where people do not have a telephone, the health worker takes it and informs the patient (Municipal health manager 7)
[Challenge 10] The use of telehealth was incipient	[...] There’s very little access to Telehealth. Although the Telehealth staff comes here [to the health region], it has already been in some municipalities, training with professionals. However, we still feel that there is still not much access [...] internet [in health units] is also a challenge (Regional manager 2). [...] we have implemented [telehealth]; however, it does not work [...] we do not have it for consulting, only for training and capacity building (Regional manager 3).

### Dimension 1 – context of health regions: socioeconomic characteristics and main flows to specialized care

In Bahia state, the health region of Ibotirama is composed of nine municipalities – among them the RRMs of Ipupiara and Morpará – and makes up the Western health macro-region, with the municipality of Barreiras as its headquarters, responsible for most of the supply of specialized care services.

The health region of Juazeiro, in Bahia state, consists of ten municipalities – among them the RRM of Pilão Arcado – and underpins the Northern macro-region of health, whose headquarters is Juazeiro, responsible for most of the provision of specialized care services. This health region borders the states of Pernambuco and Piauí.

Finally, the health region of Vale Rios Piauí and Itaueiras, in Piauí state, is composed of 28 municipalities – including the RRM of Rio Grande do Piauí – with Floriano as the regional headquarters. The largest provider of specialized care is the capital, Teresina.

The socioeconomic and demographic characteristics of the three health regions and the respective elected RRMs are summarized in Table 1.

In the four RRMs, specialized services should be distributed to include the territories’ health needs according to the health regions’ design (Fig. 1). They should be provided through the Agreed Integrated Programming (PPI). The PPI was the main tool for allocating financial resources to provide specialized care in the RRMs and was a negotiation tool between managers to decide which municipalities/public and private service providers should receive SUS financial resources to ensure specialized procedures.

Although the provider was commonly private, the most significant supply was public, especially for specialized visits and tests [Table 4, Challenge 1].

Source: National study on the organization and use of PHC services in Brazilian RRMs.

However, SUS insufficient and disorderly supply, associated with the long distances, contributed to several arrangements for the provision/acquisition of specialized care – the municipality’s direct purchase from the private provider, supply through intermunicipal agreements, and direct user disbursement (out-of-pocket expenses) – to somehow fill the care gaps or shorten the waiting time for tests [Table 4, Challenge 2].

Although the headquarters of the RRMs concentrated the primary health services, most of the population lived in rural areas in dispersed territories, thus requiring frequent travel to access some continuity between different levels of care [Table 2 and Table 4, Challenge 2].

Regarding Morpará and Ipupiara, located in the same health region, the main supply cities via PPI were Barra, Ibotirama, Barreiras, and Salvador. In turn, Irecê stood out for the supply of private services purchased directly by the municipal manager without PPI.

Concerning Pilão Arcado, the main cities of supply via PPI were Juazeiro and Salvador. Remanso also was the headquarters of the regional SAMU (Mobile Emergency Medical Service) and reference in the reading of preventive slides for cervicouterine cancer. The municipality borders the state of Piauí and has a sizeable territorial extension.

Therefore, for some rural locations, the population moved to municipalities of neighboring states, even for PHC. Because it is a city on the interstate border, Petrolina, Pernambuco state, was a vital hospital reference, especially for orthopedics.

On the other hand, in Rio Grande do Piauí, the references were concentrated in Floriano and, mainly, Teresina (state capital) [Table 4, Challenge 2].

Regarding the four RRMs, the distances between the user’s place of residence and the health care points for specialized care were the most significant organizational barriers. The issue of spatial distribution paradoxically imposed inequalities on vulnerable populations and the neediest municipalities although it met the logic of scale and scope [Table 4, Challenge 3].

Thus, the populations in rural areas of the RRMs had to travel to the municipal headquarters and then to the municipalities that provided specialized services. In this sense, the substandard road conditions, usually unpaved in rural areas, reinforced the geographical barriers to healthcare facilities.

### Dimension 2 – health care points and care regulation in RRMs: from PHC to specialized care

An emergency care facility was available at the municipal headquarters of the RRMs for urgent and emergency cases. It served to evaluate and stabilize the clinical condition, with subsequent referral to reference services. Such care units operated continuously, with the support of a team composed of doctors, nurses on duty, and auxiliary staff.

Doctors were often the same ones who worked in the PHC units, i.e., they accumulated PHC general practitioner and on-call physician duties to increase their income and, consequently, this strategy also worked as a mechanism to attract professionals. However, we observed a reduced workload in PHC units, which were deprived of doctors on duty shifts.

Some specialized public procedures were occasionally offered in the municipalities in a very segmented way.

Morpará provided visits with a psychiatrist and ultrasonography in a PHC unit, laboratory tests, and ECG in the emergency service. Informal agreements with doctors with more than one specialty were made such that, even if they were contracted for only one type of specialty, they could also support other needs, such as a psychiatrist who treated cardiology in Ipupiara.

In Ipupiara, some specialties, such as psychiatry, orthopedics, ultrasonography, and radiography were offered in the municipality through direct purchase from private doctors/clinics to compensate for the shortage via PPI.

Furthermore, the gap in the SUS table (value paid to the provider versus quantity contracted/agreed) compromised the expected supply. This situation led the municipal treasury to purchase them (inequality for poorer municipalities and more vulnerable populations).

Thus, Ipupiara complemented, at the discretion of the Health Secretary, the specialized care for users – socioeconomic and clinical criteria – who urgently needed it through direct payment to the private provider. The manager also negotiated discounts with private clinics and referred the patient who would make the direct disbursement [Table 5, Challenge 4].

Pilão Arcado offered some specialized services in the municipal hospital and complemented equally in the local private network regarding radiography, ultrasonography, and electrocardiography. Moreover, Rio Grande do Piauí offered a collection of laboratory tests in the territory. However, the laboratories were in Floriano, contracted through PPI, and offered cardiology and ultrasonography with their resources.

Thus, in SUS vacuum, specialist doctors went to the RRMs and offered visits, procedures, and tests for user’s direct payment or sold them to the municipal public entity. Such private offers were intermittent and residual in the set of needs of the population, mainly due to the socioeconomic conditions of most inhabitants and RRMs’ budget constraints. However, the municipal manager’s direct purchase seemed to stimulate the private network [Table 5, Challenge 5].

Another contradiction due to the long trips to the provider was that it was sometimes more advantageous for users to acquire the procedure/visit by direct disbursement when offered by the private initiative in their city of residence or the closest municipality. Not coincidentally, the RRMs, when possible, offered procedures in their territory or bought from neighboring providers outside the PPI because it was less expensive than having health transportation and, in some cases, accommodation. This organizational rationale strengthened the private provider and split the network modeling. For example, Rio Grande do Piauí’s management collected laboratory tests weekly in the municipality and sent them to Floriano to be analyzed to minimize such problems, thus avoiding users’ commuting. [Table 5, Challenge 6].

The lack of vacancies for specialized care was a reality in all three health regions. It affected all the municipalities indiscriminately; however, they seemed to be more harmful to the RRMs since they concentrated more significant difficulties of geographical access and more vulnerable populations.

As specialized care and long-term care offers are often located in the respective state capitals, all the RRMs provided patients with a support house for their stay. The users received the Out-of-Home Treatment (OHT) benefit. However, the total amount of the federal program transfer to the municipality did not meet the need and managers supplemented most of it with their monies [Table 5, Challenge 7].

In all municipalities, the appointment scheduling centers centralized in the health secretariats mediated access to specialized care. Information about the appointment scheduling period was often provided informally to the population. The community health worker (CHW) was essential in providing information on appointment scheduling and the results of specialized tests, especially in rural areas [Table 5, Challenge 8].

When users managed an appointment for specialized care, patients brought with them the clinical information since there was no integrated information system. One of the few exceptions was the high-risk prenatal care in Ipupiara, with flow and counterflow between the municipality and the hospital in Barreiras.

The counter-referral system was unreliable, and the patients were the primary informants about their health conditions and history of visits. The community health workers shared this information with the PHC team (hospital discharge, the performance of procedure, and therapeutic plan) [Table 5, Challenge 9].

Despite the enormous difficulties in access to specialized care and no health transport in any of the RRMs, telemedicine was systematically employed and the units were partially computerized. Also, telephone and internet communication services were unreliable, especially in the rural areas, i.e., contrary to the needs of remote territories [Table 5, Challenge 10].

## Discussion

In the three health regions studied, the frayed regional logic of the SUS and the impasses of the care gaps compromise comprehensive health care for the population of the RRMs, resulting in inequalities since these are the most impoverished municipalities with the most vulnerable population.

The managerial, financial, and technical rationality imposes the concentration of health equipment for specialized care in cities that are reference points for several other municipalities [21, 36], although inter-municipal cooperation is motivated primarily by the economies of scale and cost efficiency [37, 38].

The RRMs lack the rationality – scale and scope – required to assume regionalized health services networks. As a result, even when covered by specialized public services, the population commonly purchase health services – visits, tests, or procedures – by direct disbursement, given the barriers of geographical access to public services, such as long distance and shortage of health transportation, and greater availability of private providers nearby.

Given this reality, the managers seek to offer specialized public care in their territory through direct purchase with professionals or private clinics in a segmented and unilateral way, to the detriment of intermunicipal regional planning.

However, these are tiny and poor municipalities, which means that spending health care directly with private providers with own revenues of the municipal treasury reduces the bargaining power of public managers and exposes them to the deregulated market game – in price, quantity, and quality of the product offered – signaling fiscal inequalities [39, 40].

The historical absence of social policies in rural territories [22, 41, 42] has subjected them to all sorts of exploitation as clients [43] and selective programs aimed at appeasing specific problems without reversing persistent inequalities [42].

To some extent, the health services reflect these discrepancies and, therefore, the RRMs accumulate different needs that, in synergy, increase the health demands. Nevertheless, the monopoly of services and medical specialties [44], the autonomy of the physician [45], and the asymmetric diagnostic conduct [46] submit the population and often managers to the shackles of biomedical rationality [47], not always committed to the production of health care [48].

The RRMs struggle in attracting and retaining professionals [49], contributing to the lack of care at the first level of the network and the accumulation of acute diseases to PHC [50]. On the other hand, the specialized services (scarce and concentrated) are pressured by a demand far beyond what is routinely expected, as it emerges from a population whose diseases are diagnosed late, aggravated by the lack of timely care, faced with very unfavorable socioeconomic issues, and simultaneously affected by a triple burden of disease [13]. Therefore, this population requires comprehensive and continuous public policies to reverse social inequalities in health through intersectionalities [51, 52]. Moreover, all the problems identified are worse for populations residing in rural areas of the RRMs, i.e., there is a need for specific health policies that reach vulnerable population groups within territories already at a socio-spatial disadvantage vis-à-vis the surroundings.

In contrast, the modeling of health regions has sustained unassisted care and encouraged the private market for selling visits and isolated procedures precisely in impoverished territories [9]. This paradoxical effect of regionalization [53] reflects the federative mechanism of the country since the municipal manager can define the place of service implementation.

However, the financial cap and the availability of providers [38] impose organizational restrictions. Also, in the rationale of sharing services among municipalities, the more rural and remote the territory is, the more it will require the health procedure, provide health transportation, support homes, and other logistical actions to facilitate user travel or permanence during the period required for treatment away from home [54].

In any case, RRMs can benefit from investments in health in same-health-region cities because health expenditures have significant spatial externalities [55]. In this sense, synergistically, the municipal health expenditure reduces the hospitalization of residents in neighboring smaller municipalities and curbs the demand for hospitals in neighboring larger municipalities [55].

Thus, health problems are not overcome through specialized services nor exclusively through access to them. The lack of network integration, aggravated by segmented supply [56], imposes the lack of a communication relationship between PHC and specialized care professionals [57]. Such precarious communication in RRMs begins with scheduling appointments concentrated in the municipal health secretariats and PHC professionals’ total lack of knowledge of the size and clinical profile of users on the waiting list for specialized care. All these issues compromise quality health care [32, 58].

Each user/household is responsible for the flow of clinical information between professionals (counter-referral), resulting in information amnesia that implies recurrent clinical rework, aggravated by the turnover of PHC doctors, overlapping therapeutic behaviors, and the loss of contact for care continuity [59]. Another significant aspect is that some users with serious diseases end up “losing” contact and trust in the first level of care professionals and link to other care, resulting in the detriment of care continuity and coordination via PHC [60].

Not coincidentally, in the vacuum of information technologies in RRMs, community health workers are the strategic informants and keep the PHC team informed, albeit not systematically, of the users’ flows to the specialized care, relying, once again, on the memories of patients and family members.

A final element is the underutilization of telehealth strategies, a strategic resource in remote areas. In countries like Australia, telehealth is even used for specialized visits, accessed directly by the user [20]. Developing telemedicine strategies in remote areas can facilitate access to specialized care, prevent avoidable hospitalizations [20, 61], and should be the subject of investment in the country [62].

Such notes also imply looking at the near future and the challenges for the SUS before the Brazilian epidemiological transition, which differs from that observed in high-income countries [63, 64].

Although chronic non-communicable diseases (NCDs) are responsible for the largest share of the disease burden in the country, including RRMs, infectious diseases, and external causes coexist [65]. The triple burden of disease reflects Brazilian social inequalities expressed, among other factors, in access to health goods and services [13, 64].

The complex nature of the epidemiological situation [63] and population aging [66] require implementing integrated health care networks [67] coordinated by PHC^59^ in regional territories [60], in which specialized care can be accessed from a sociocultural, epidemiological, and clinical perspective, without exposure to unnecessary procedures or mitigation of the full right to health guaranteed by the SUS [65].

Moreover, COVID-19 and its health consequences [68] – including sequelae – deep social inequalities and food insecurity – more incident in highly impoverished areas (for example, RRMs) – mental health problems, and the progressive growth of chronic degenerative diseases [64], some related to the aging population [66], should pressure the demand for specialized care.

In this context, we should rethink the health care structures and model. The scarce supply of specialized services and timely diagnostic support have been enhanced to some extent by the current biomedical model, which has also occurred in the RRMs. This situation reduces PHC resolution and makes it a producer of demands. Therefore, revaluating PHC professionals, such as increasing the number of nurses per team, engaging the oral health team in all health units, and increasing the number of CHWs, against the established in the latest national policies, are primary requirements to expand the clinical scope, reduce the excessive dependence on medical practice, and curb the demand for specialized services. Furthermore, it is fundamental to recognize and incorporate community care and local/traditional practices into the therapeutic menu to develop sustainable care modalities and adopt an intercultural approach. All these factors corroborate the need to change health care’s technological core.

An important limitation of the study is that the constellation of interviewed subjects can be expanded per the desired focus and capture perspectives that respond to the different aspects to understand the challenges in the organization and provision of specialized care. Qualitative research is insufficient to unveil all the obstacles to the assumption of a resolution-based regionalized network and requires other approaches to expand the scope of data to enable the triangulation of methods. Furthermore, we consider that, in future research, patients and their families could and should provide their health and treatment histories in the RRMs and, thus, expand the dialogue with different stakeholders who use health services daily.

## Concluding remarks

Integrating services with the regional network is quite fragile in the RRMs. Most of the time, they are only the financier, leaving the provider and regulatory functions in the hands of reference municipalities. In this area, the incipiency or lack of communication tools between care levels compromises the attribute of coordination. In this sense, it favors inadequate diagnoses and treatments with severe consequences to patient safety.

Thus, although the spatial externality of health expenditure can benefit the municipalities in the vicinity of the investment, local governments – responsible, predominantly, for PHC in their territories – require technical and financial cooperation from the federal and state governments to secure a resolute PHC integrated into the care network, generating reciprocal gains for the entire health region.

Finally, historically unassisted (small, rural, and remote) municipalities remain in a vicious circle between poverty that generates illnesses, including preventable ones, and the technical-economic insufficient supply of services to treat and prevent them, enduring or generating new illnesses and exacerbating existing problems.

From this perspective, globally universal health systems require sophisticated strategies to ensure comprehensive care that cannot be restricted to the supply of services since a range of socio-sanitary specificities implies access barriers.

We propose some actions that health managers and policymakers can consider to improve access to specialized services for populations in remote rural territories, such as computerization and connectivity of PHC units (computers, mobile devices, and internet); expanding and enabling the use of remote services via the internet – telediagnosis, tele-visit with a specialist, telemonitoring of health conditions and patient surveillance, second opinion with a specialist and telehealth for in-service professional education; a federal policy for the procurement of health transport (different types of vehicles adapted to the conditions of unpaved roads) and financial resources for vehicle maintenance; a federal policy with co-financing (capital resources and costing) to expand the offer of specialized public services, via the Intermunicipal Health Consortium in strategic municipalities in the health regions; a state-funded regional policy that enables visit with a PHC specialist in RRMs or itinerant specialist (Mobile Units with Experts); use of portable diagnostic equipment in PHC units; regional articulation of all points of provision of specialized services distributed across the different municipalities and streamlining the integrated offer in the health region; cooperation between states, especially in support of border municipalities; and national policy to encourage the training and internalization of expert physicians.

To this end, specialized public services must be provided in the region and made accessible, primarily due to the historical care vacuum in the vicinity of the RRMs. However, health policy should focus on reducing demand; thus, investment in high-quality PHC is a priority.

In short, all the possibilities mentioned may possibly be implemented together to eliminate inequalities, although they are not enough given the socio-health complexity of the territories studied. Therefore, the current and persistent socioeconomic inequalities experienced by populations living in RRMs must also be considered and comprehensive cross-sectoral policies will be critical to achieve a successful and lasting change.

## Data Availability

The data analyzed during the current study are not publicly available because it would infringe on the confidentiality agreement with the study participants. Research tools can be shared upon request to the corresponding author.

## Abbreviations

CHW: community health worker
MM: Municipal Health Secretaries and Primary Health Care Coordinators
OECD: Organization for Economic Co-operation and Development
OHT: Out-of-Home Treatment
PHC: primary health care
PPI: Agreed Integrated Programming
RM: regional managers
RRM: remote rural municipalities
SAMU: Mobile Emergency Medical Service
SM: state managers
SUS: Unified Health System
